# Vagotomy attenuates bleomycin-induced pulmonary fibrosis in mice

**DOI:** 10.1038/srep13419

**Published:** 2015-08-20

**Authors:** Nana Song, Jun Liu, Saad Shaheen, Lei Du, Mary Proctor, Jesse Roman, Jerry Yu

**Affiliations:** 1 Robley Rex VA Medical Center, Louisville, KY, USA; 2 Division of Pulmonary, Critical Care and Sleep Disorders, Department of Medicine, University of Louisville, Louisville, KY, USA; 3 Department of Pharmacology and Toxicology, University of Louisville, Louisville, KY, USA; 4 Department of Physiology and Biophysics, University of Louisville, Louisville, KY, USA

## Abstract

The progression of pulmonary fibrosis (PF) entails a complex network of interactions between multiple classes of molecules and cells, which are closely related to the vagus nerve. Stimulation of the vagus nerve increases fibrogenic cytokines in humans, therefore, activation of the nerve may promote PF. The hypothesis was tested by comparing the extent and severity of fibrosis in lungs with and without vagal innervation in unilaterally vagotomized mice. The results show that in vagotomized lungs, there were less collagen staining, less severe fibrotic foci (subpleural, peri-vascular and peri-bronchiolar lesions) and destruction of alveolar architecture; decreased collagen deposition (denervated vs intact: COL1α1, 19.1 ± 2.2 vs 22.0 ± 2.6 ng/mg protein; COL1α2, 4.5 ± 0.3 vs 5.7 ± 0.5 ng/mg protein; p < 0.01, n = 21) and protein levels of transforming growth factor beta and interleukin 4; and fewer myofibroblast infiltration (denervated vs intact: 1.2 ± 0.2 vs 3.2 ± 0.6 cells/visual field; p < 0.05, n = 6) and M2 macrophages [though the infiltration of macrophages was increased (denervated vs intact: 112 ± 8 vs 76 ± 9 cells/visual field; p < 0.01, n = 6), the percentage of M2 macrophages was decreased (denervated vs intact: 31 ± 4 vs 57 ± 9%; p < 0.05, n = 5)]. It indicated that the vagus nerve may influence PF by enhancing fibrogenic factors and fibrogenic cells.

Pulmonary fibrosis (PF) is a devastating disease. Although the role of sundry cellular and immune modulator factors has been intensely investigated, the pathogenesis of PF remains idiopathic in most cases. Previous investigations have implicated multiple cell types [fibroblasts, myofibroblasts and M2 macrophages activated alternatively by T helper-2 (Th-2) cells] and cytokines [transforming growth factor β (TGF-β) and interleukin 4 (IL-4)]. TGF-β, a regulator of cell proliferation and differentiation, is a key fibrogenic cytokine. It modulates fibroblast phenotype and function, inducing myofibroblast transdifferentiation and promoting matrix preservation^1^. IL-4, a multi-functional cytokine playing significant roles in immune cellular regulation including chemotaxis, is also fibrogenic, which promotes alternative activation of macrophages into M2 cells (“repair macrophage”) that leads to increased production of TGF-β, thereby leading to myofibroblast differentiation and increased fibrosis^2^. Both TGF-β and IL-4 increase in bleomycin-induced lung injury^3^,^4^. Myofibroblasts, identified by expression of α-smooth muscle actin (α-SMA), are the primary source of type I collagen in fibrotic diseases^5^. The presence of myofibroblasts in fibrotic sites in lung tissues, both from humans and animals with PF, is well documented^6^,^7^. In addition to IL-4 and TGF-β, M2 macrophages are pro-fibrogenic^8^,^9^,^10^. In both bleomycin-induced lung injury and TGF-β over-expressing mouse models, PF can be prevented by phenotypic modulation of macrophages^11^,^12^.

Recently, the vagus nerve has been found to play an important role in neuro-immune interaction and is involved in a variety of pulmonary disease processes. However, its relation to PF is not known. Many reports suggest that the vagus nerve may be directly or indirectly involved in fibrogenic processes. For example, stimulation of the vagus nerve in patients significantly increased plasma levels of TGF-β^13^. Vagotomy eliminated drug-induced activation of TGF-β in the cerebral spinal fluid^14^. Cholinergic denervation decreased TGF-β1 expression in CCl4-induced liver fibrosis in rats^15^. Also, neuropeptides may alter T-cell responses, switching a Th-1 response to a pro-fibrogenic Th-2 response. However, we are not aware of any study that directly investigates the role of the vagus nerve in the development of PF. We hypothesize that activation of the vagus nerve enhances PF (Fig. 1). Specifically, if our hypothesis is correct, vagotomy should attenuate PF. To test the hypothesis, we compared bleomycin-induced fibrosis in lungs with or without vagal innervation in the same mouse, and found that vagal denervation ameliorated PF. The protective effect of vagotomy was further confirmed by decreased production of cytokines (TGF-β and IL-4) as well as myofibroblast proliferation and M2 macrophage differentiation.

## Results

### Even blood supply to vagotomized and intact lungs

Prior to establishing our murine vagotomy model, we needed to rule out the possibility that vagotomy may reduce blood supply to affect lung fibrosis. Therefore, we compared the blood distribution in both lungs of unilateral vagotomized mice and found that the blood supplies per unit lung tissue were the same. The ratio of EB concentration (vagotomized over intact lungs) was 98.6 ± 1.8% (n = 8).

### Vagotomy decreased collagen deposition and preserved lung architecture

**Figure 2:**
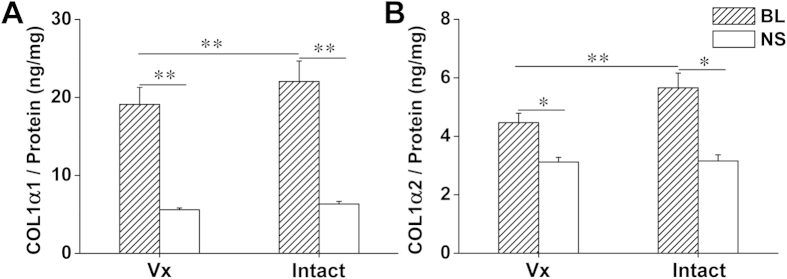
Collagen production in the lung. Protein levels of COL1α1 (**A**) and COL1α2 (**B**) were increased 4 weeks after bleomycin treatment with greater increases in the intact lung. BL: bleomycin (n = 21); NS: normal saline (n = 6); Vx: vagotomy. *P < 0.05, **P < 0.01.

**Figure 3:**
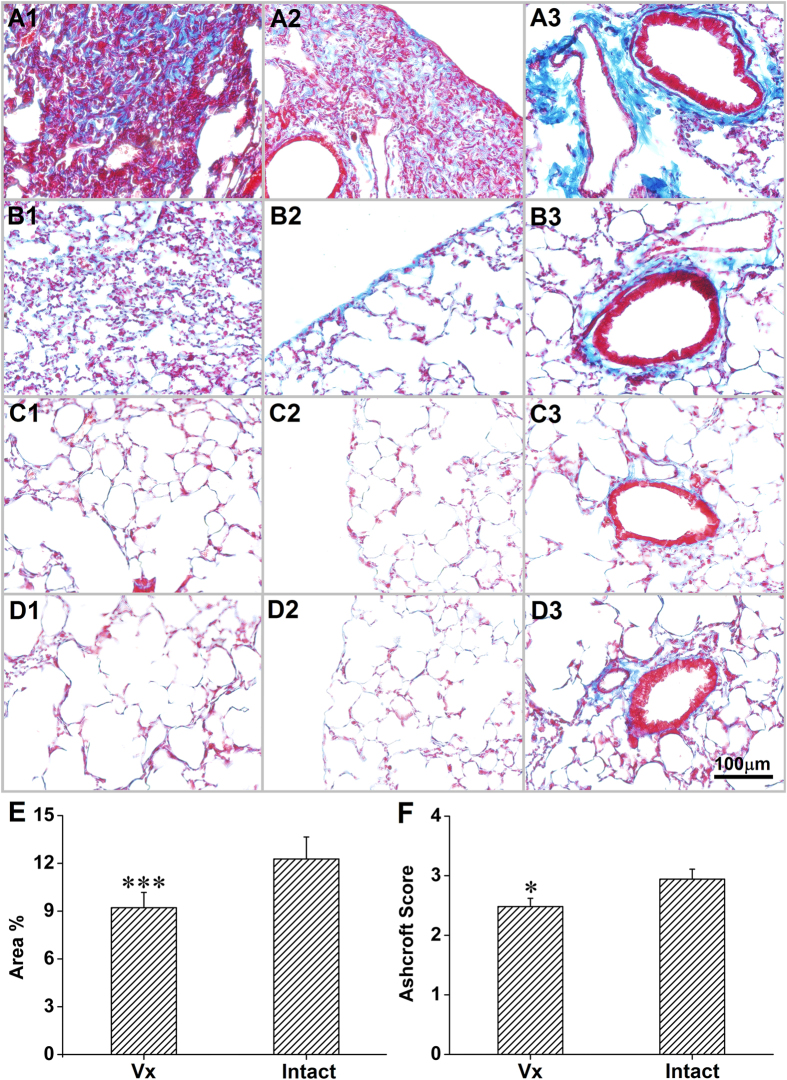
Evaluation of collagen deposit in innervated (**A**,**C**) and denervated (**B**,**D**) lungs by Masson’s trichrome stain. a and b, bleomycin-treated group; c and d, vehicle control group. Following bleomycin, there were more lesions in the intact lung, demonstrated by 1, consolidation of lung parenchyma with loss of alveolar architecture and increased cellularity; 2, subpleural lesions; 3, peri-vascular and peri-bronchial collagen deposits. (**E**) morphometric analysis shows that collagen deposit was higher in the intact lung. (**F**) the Ashcroft score was higher in the intact lung. Vx: vagotomy. *P < 0.05, ***P < 0.001, n = 27.

**Figure 4:**
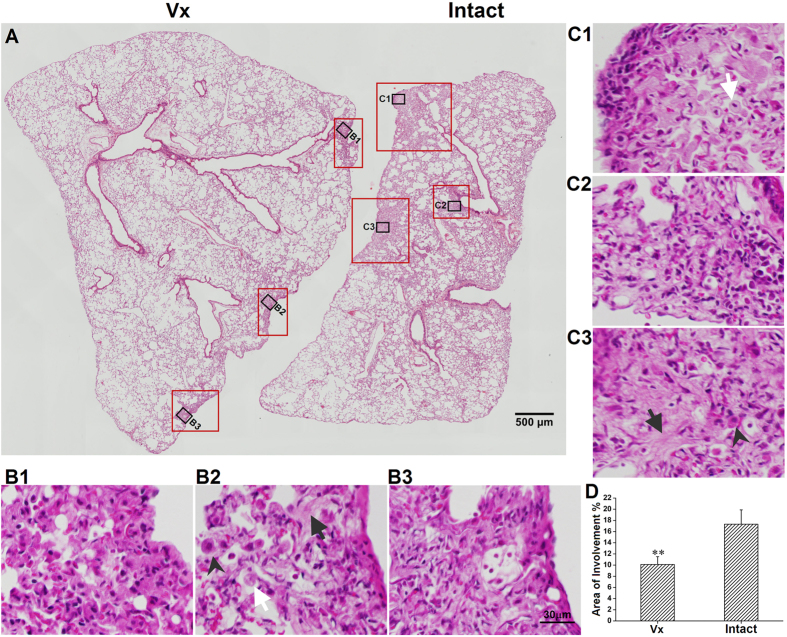
Evaluation of lung parenchyma lesions following bleomycin challenge by H&E stain. (**A**), gross view. (**B1**,**B2**,**B3**) and (**C1**,**C2**,**C3**), represented high power visual field of area of involvement, showing detailed consolidation in fibrotic areas from the intact right and denervated left lungs, respectively. Arrow shows young collagen, white arrow shows macrophage, arrow head shows the type II alveolar epithelial cell. (**D**) morphometric analysis shows that percent consolidation area was more in the intact lung than the denervated lung. Vx: vagotomy. **P < 0.01, n = 24.

Four weeks after bleomycin treatment, collagen deposition was increased more in the intact lung than the vagotomized lung, evidenced by increased expression of COL1α1 and COL1α2 (ELISA) (Fig. 2), and by increased fibrosis in the Masson’s trichrome stain. The percentage of stained collagen area (per cross sectional area) and Ashcroft scores were significantly greater in the intact lung than the vagotomized lung (Fig. 3). In addition to fibrosis, there was increased damage to alveolar architecture. Fibrotic lesions were mainly subpleural, peri-vascular and peri-bronchial. Typical fibrous foci, distortion of lung architecture, interstitial hyperplasia and inflammatory cell infiltration were observed in the H&E stain (Fig. 4). The total area of involvement was lower in the vagotomized lung (Fig. 4D).

### Vagotomy decreased the production of bleomycin-induced fibrogenic cytokines

**Figure 5:**
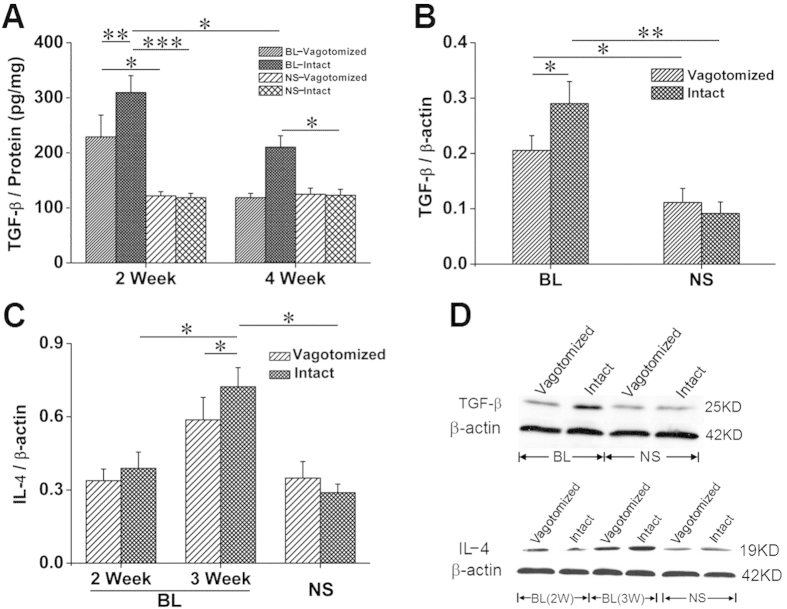
Protein expression of pro-fibrogenic cytokines in the lung. (**A**) two weeks after bleomycin injection, total TGF-β (ELISA) increased with more in the intact lung (BL, n = 6; NS, n = 5) and so TGF-β activity (Western blot) (**B**, n = 8). (**C**) three weeks after bleomycin injection, IL-4 (Western blot) increased with more in the intact lung (BL, n = 4; NS, n = 5). (**D**) representative Western blots for TGF-β and IL-4. *P < 0.05; **P < 0.01; ***P < 0.001.

Two weeks after bleomycin treatment, levels of total (latent and activated) TGF-β1 (ELISA) as well as activated TGF-β1 (Western blot) were higher in the intact lung than in the denervated lung. Although the level of TGF-β1 decreased in both lungs, it remained significantly higher in vagus-intact lungs at 4 weeks. Similarly, IL-4 production was also increased to a greater degree in the intact lung (Fig. 5).

### Vagotomy suppressed proliferation of fibrogenic cells

**Figure 6:**
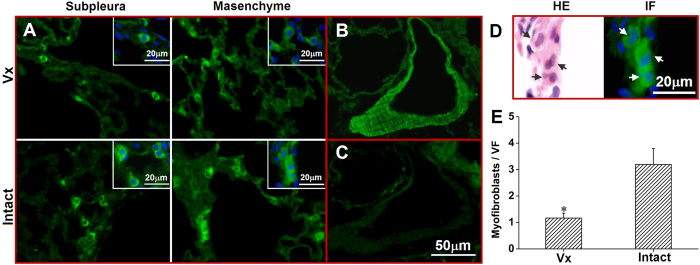
Myofibroblasts in the lung. (**A**) there were more myofibroblasts (green α-SMA positive cells) located in subpleural area and lung interstitium in the intact lung. (**B**) stain of vascular smooth muscle serves as a positive control; (**C**) negative control (no primary antibody); (**D**) A representative morphology of myofibroblasts in both H&E and IF stains; (**E**) group data (n = 6, *P < 0.05). Vx: vagotomy.

**Figure 7:**
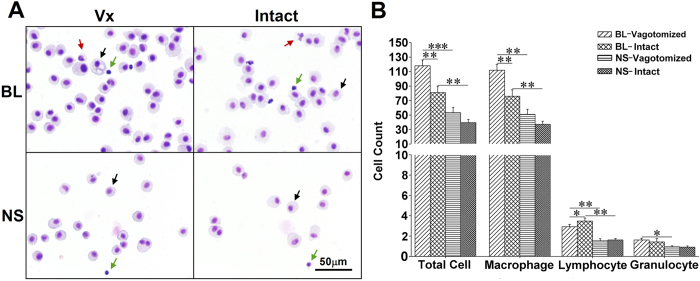
Cell differentiation in BALF with the Diff-quick method. (**A**) representative visual fields. Cells were primarily macrophages (black arrows) with a few lymphocytes (green arrows) and granulocytes (red arrows) 4 weeks after bleomycin treatment. (**B**) group data show that cell number in both lungs increased with a greater number of macrophages and lower number of lymphocytes in the vagotomized lung after bleomycin treatment. BL, bleomycin; NS, normal saline; Vx, vagotomy. *P < 0.05; **P < 0.01; ***P < 0.001; n = 6 in BL group, n = 4 in NS group.

**Figure 8:**
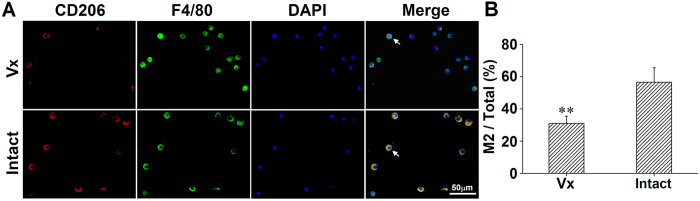
Macrophages in BALF slides 4 weeks after bleomycin treatment. (**A**) visual fields show that cells were primarily macrophages (F4/80^ +^). The number of macrophages was greater but M2 (CD206^ +^/F4/80^ +^) was lower in the vagotomized lung. (**B**) group data show that percentage of M2 over the total number of cells was lower in the vagotomized lung. Vx: vagotomy. n = 5, **P < 0.01.

Myofibroblasts (α-SMA-positive cells) in lung sections and CD206 ^+^/F4/80 ^+^ cells in BALF smears were counted. There were increased number of α-SMA positive myofibroblasts and M2 (CD206 ^+^/F4/80 ^+^) macrophages per visual field in the intact lung. Myofibroblasts were mainly located in the subpleural areas and within fibrous lesions (Fig. 6). Diff-quick stain showed mainly macrophages/monocytes in BALF. The total number and type of leukocytes (i.e., macrophage, lymphocyte and granulocyte) were analyzed. Bleomycin challenge increased cell numbers with more cells detected in the vagotomized lungs (8.4 ± 3.5 × 10^4^/ml) than in the vagus-intact lungs (5.5 ± 3.0 × 10^4^/ml; n = 22, p < 0.001). However, there were more lymphocytes in the intact lung (Fig. 7). Although M2 macrophages were only slightly higher, the ratio of M2/M1 macrophages was significantly higher in BALF from the intact lung (Fig. 8).

## Discussion

We established an animal model to assess the role of the vagus nerve in the pathophysiological process of PF by comparing bleomycin-induced fibrosis in lungs of unilaterally vagotomized mice. Difficulties are encountered when interpreting data generated to assess the roles of the vagus nerve in lung diseases by comparing vagotomized with vagus intact animals, mainly because the nerve not only innervates the lung, but also other organs such as the heart and digestive system. It also transmits vital information to the brain, which involves neuroimmune interaction^16^,^17^. Clearly, vagotomy affects the function of multiple organs and interpretation can be difficult. However, our newly established, unilaterally vagotomized mouse model enables us to directly compare innervated vs denervated lungs in the same animal. Thus, the effects of the vagus nerve on PF can be investigated. In models where bleomycin is administered using the intra-tracheal route, we cannot compare the left with the right lungs in the same animal because the amount of bleomycin reaching each lung is unpredictable. In order to solve the problem, we used an intravenous injection model, in which bleomycin is evenly distributed. In this scenario, the severity of lung involvement in right and left lungs can be compared in the same animal. However, it can be argued that vagotomy may leave sympathetic vasoconstriction unopposed, which might negate drug delivery by an intravenous injection. To rule out this possibility, we performed experiments to compare the blood distribution in both lungs of unilaterally vagotomized mice and found that the blood supply per unit lung tissue was the same for both lungs.

Having characterized the mouse model, we engaged in studies evaluating the role of the vagus nerve in bleomycin-induced PF. Our data support the hypothesis that the vagus nerve influences bleomycin-induced PF by demonstrating that vagotomy: 1) decreased collagen deposition; 2) attenuated structural destruction; 3) reduced production of pro-fibrogenic cytokines; and 4) suppressed pro-fibrogenic cells.

In the lung, type I collagen, the main fibril collagen, consists of two α1(I) and one α2(I) chains^18^. Both COL1α1 and COL1α2 subunits are increased in the bleomycin-induced PF mouse model^19^. Although different in methodology, our results parallel prior findings of increased COL1α1 and COL1α2 subunits (Fig. 2). Interestingly, vagotomy reduced the increased COL1α1 and COL1α2 by approximately 30%. Masson’s trichrome stain shows a similar decrease in collagen stain in the vagotomized lung (Fig. 3).

Histologically, PF is characterized by an increase in inflammatory cells, fibroblasts, and collagen deposition, and by alterations in alveolar architecture with marked regeneration and remodeling^20^. In our studies, mice developed classic PF with structural alteration of the lung following intravenous bleomycin treatment (Figs 3 and 4). Lung lesions were apparent but not severe, as indicated by the low Ashcroft scores. Vagotomy attenuated structural damage and alleviated fibrosis. This is supported by significantly lower Ashcroft scores in the vagotomized lung.

Among various fibrogenic cytokines, TGF-β and IL-4 are the most critical. TGF-β1 production increases in patients with idiopathic pulmonary fibrosis (IPF) and in animal models of PF. TGF-β promotes epithelial-mesenchymal transition and fibroblast transdifferentiation (transforming resident fibroblasts into myofibroblasts)^21^. TGF-β and IL-4 act in concert to play a crucial role by promoting macrophage polarization towards the M2 phenotype. IL-4 regulates fibroblast function as well as myofibroblast differentiation^22^. In human studies, progression of IPF is associated with augmented IL-4 production^23^. IL-4-/- mice develop significantly less PF after treatment with bleomycin^24^. Moreover, IL-4 stimulates TGF-β production in a mouse model of skin fibrosis^25^. In our studies, levels of TGF-β and IL-4 were higher in innervated lungs than denervated lungs (Fig. 5).

The fibrogenic process engages both resident and recruited myofibroblasts and M2 macrophages. Myofibroblasts are characterized by the formation of a contractile apparatus (α-SMA microfilaments) and secretion of extracellular matrix components (type I collagen). Induction and maintenance of the M2 macrophages, which express CD206 (macrophage mannose receptor), is a characteristic feature of PF^26^. Our results show that the number of myofibroblasts and the percentages of CD206 + cells in macrophages (F4/80 + cells) were higher in the vagus-intact lung. Furthermore, lymphocytes, a key source of IL-4, were more numerous in the intact lung than the vagotomized lung.

The role of inflammation in IPF is controversial. Fibrosis may occur without inflammation and anti-inflammatory or immunosuppressive therapy has not been effective for IPF^27^. However, inflammation is part of the pathological process in bleomycin-induced PF animal models. This is evidenced by a significant increase in the number and percentage of lymphocytes in BALF following intra-tracheal instillation of bleomycin^28^. In the present study, we also found that inflammatory cells increased after intravenous treatment with bleomycin. However, cellular alterations in BALF appeared to be inversely related to severity of PF (i.e. the vagotomized lung had more inflammatory cells but less fibrosis). It needs to be emphasized that the percentage of lymphocytes was lower in vagotomized than intact lungs. This is consistent with a human study showing that T lymphocytes are increased in the areas of interstitial fibrosis and worsen PF^29^.

We should highlight the fact that our results show that the vagus nerve does not drive the development of PF, but modifies it, because PF develops with or without vagus innervation. The degree of fibrosis, as measured by the ratio of area of fibrosis to total lung area, is 25% higher in vagus intact lungs than vagotomized lungs. Vagotomy reduced bleomycin-induced collagen deposition by 30%. This effect is not trivial and is comparable with the effect of blocking TGF-β, one of the most important fibrogenic molecules. For example, injection of anti-TGF-β antibody into the mouse was able to neutralize the fibrogenic effect of TGF-β by virtue of a 40% reduction in collagen accumulation^30^. Neutralizing TGF-β with a chimeric soluble receptor decreased lung hydroxyproline by 33%^31^. Epithelium-specific deletion of TGF-β receptor type II attenuated collagen production by 50%^32^. Compared to these studies, it appears that the vagus nerve is a powerful modulator in the development of PF.

Bleomycin, an antibiotic, is a cytotoxic agent used to treat a variety of neoplasms. Its cytotoxicity occurs mainly in the lung. Thus, it is commonly used for studying human PF in C57BL/6. Following bleomycin treatment the lung undergoes significant biochemical, histological and physiological changes, which are similar to those of humans, leading to PF. Thus, this model can provide useful insights into the mechanisms of lung injury, repair, and fibrosis. However, the fibrosis in the mouse resolves. Thus, direct extrapolation of mouse data into humans is dangerous. Furthermore, our current intervention only substantiates a potentially effective preventative strategy, however, whether it can be used as a treatment procedure awaits verification.

One concern is that vagotomy lacks clinical application. However, we use vagotomy as a tool to examine the neural mechanisms in PF. Our data demonstrate that removing the nerve actually ameliorates bleomycin-induced fibrosis. Therefore, further investigation to delineate effects of vagal afferent and efferent nerves is warranted. More questions need to be addressed regarding specific neural transmitters and peptides involved in the observed effects.

In summary, we have established a mouse model to assess the role of the vagus nerve in the development of PF. Our data demonstrate that the vagus nerve is a modulator for PF development. Since vagotomy decreases deposition of collagen, we conclude that activation of the vagus nerve promotes PF. This is further confirmed by the decreased numbers of fibrogenic cells (myofibroblasts and M2 macrophages) and decreased production of fibrogenic cytokines (TGF-β and IL-4) in the vagotomized lung.

## Methods

### Animals

Male C57BL/6J mice (The Jackson Laboratory, Bar Harbor, ME, 20 weeks, 30–40 g) were housed 4 in one acrylic cages with shredded corn cob bedding in an acclimatized room (12/12 h light/dark cycle; 22 ± 3°C) and provided with water and mouse breeder chow ad libitum, according to standard protocols for animal care. The procedures used were approved by the Institutional Animal Care and Use Committees of the University of Louisville and the Robley Rex VA Medical Center, in compliance with the United States Public Health Service Standards and National Institutes of Health guidelines.

### Mid-cervical vagotomy

To eliminate potential difference between the two lungs we randomly divided 50 mice into left vagotomy (LV) and right vagotomy (RV) groups. After anesthesia with 3% isoflurane using a veterinary anesthesia system, left or right vagus nerve (a minimum of 5 mm) was isolated and sectioned at the mid-cervical level^33^. Analgesic was provided for two days. Two weeks were allowed for recovery before bleomycin treatment.

### Bleomycin treatment

Mice were anesthetized with 3% isoflurane and injected with bleomycin via the tail vein (80 mg/kg body weight, dissolved in 200 μl of 0.9% NaCl solution)^34^. For control mice, 200 μl of 0.9% NaCl solution was injected. Body weight was measured every other day. Mice were sacrificed in 2, 3 and 4 weeks.

### Blood distribution assessment

Unilaterally vagotomized mice were anesthetized and injected with Evans Blue (EB, 50 mg/ml at 50 mg/kg) via the tail vein. Lung tissues were harvested 10 minutes after injection and oven-dried. EB in the tissue was extracted in formamide (50 ml/g tissue). EB concentrations were measured with a spectrophotometer at a wavelength of 620 nm.

### Morphological examination

The lung was fixed by intratracheal infusion of 4% paraformaldehyde and then immersed in fixative overnight. Transverse sections from each side of the lung were embedded in paraffin and 5 μm sections were prepared and stained with haematoxylin-eosin (H&E) and Masson’s trichrome. The intact image of total lung transverse slice was scanned with an Olympus microscope. Fibrotic lung injury was assessed by Ashcroft scoring and quantitative analysis by two investigators blinded to tissue samples.

#### Ashcroft scoring

Each side of the lung was individually assessed for severity of interstitial fibrosis and given a score between 0–8 with the Ashcroft scoring system as described: grade 0-normal lung; grade 1-minimal fibrous thickening of alveolar or bronchiolar walls; grade 3-moderate thickening of the walls without obvious damage to lung architecture; grade 5-increased fibrosis with definite damage to lung structure and formation of fibrosis or small fibrous masses; grade 7-severe distortion of lung structure and large fibrous areas; grade 8-total fibrous obliteration of the field^35^. Difficulty in deciding between odd-numbered categories was resolved by giving the intervening even-numbered grade.

#### Quantitative analysis

(a) Collagen fraction: the degree of fibrosis was quantified with the photoshop image program as previously described and by adjusting the threshold of color settings^28^. The image analysis program was configured to detect areas of blue-stained collagen within each lung transverse section stained by Masson’s trichrome. By reverse selection of the background, the software calculated the area of lung parenchyma. The fractional area of collagen was expressed as a percentage of the total area of lung parenchyma. (b) Lung involvement fraction: Lung involvement was defined as definite distortion of lung architecture^36^. Using the cellsens dimension program, areas of lung involvement in the total lung section were circled and calculated by the polygon method. Lung involvement fraction was expressed as a percentage of the total area of lung sections.

### Bronchoalveolar lavage

The left and right lungs were separated and lavaged^37^. A tracheal cannula attached to a 1 ml syringe was used to lavage three times [1.2 ml (0.4 × 3) aliquots of 1 × HBSS without Ca^2 +^ or Mg^2 +^ for the right lung and 0.6 ml (0.2 × 3) for the left lung]. Lavage cells were collected, and centrifuged at 200 g for 5 minutes at 4 °C. The cells from the right and left lungs were suspended in 1.2 ml and 0.6 ml fresh HBSS, respectively and centrifuged onto glass slides, and stained with a Diff-Quick Staining kit (Fisher Scientific, MA, USA ). The total number and classification of cells from both sides of the lung were counted and analyzed separately.

### Immunofluorescence staining

Tissue sections were deparaffinized and rehydrated using sequential xylene and ethanol rinses. After antigen retrieval in 0.01 M citrate buffer, pH6.0, 98 °C for 15 min (bronchoalveolar lavage fluid (BALF) was smeared and fixed in methanol), the sections were pre-incubated with 5% normal goat serum to block nonspecific binding and then incubated with primary antibodies (rabbit anti-mouse α-SMA, goat anti-CD206 antibody and FITC conjugated rat anti F4/80 antibody) at 4 °C overnight. Extensive washing was followed by FITC-conjugated goat anti-rabbit IgG or cy3-donkey anti goat IgG (1:200) for 1 h at room temperature. The numbers of α-SMA-positive cells in lung sections and CD206^ +^/F4/80^ +^ cells in BALF smears were counted at 200 × magnification in lung specimens obtained from each lung side (n = 3 in each group). Six fields from each side were counted. Positive cell counts and proportions were expressed as the average number of cellular profiles per field.

### Western Blotting

Lung tissue samples containing 20 μg protein were electrophoresed upon polyacrylamide gel and electro-blotted onto nitrocellulose membranes. Then, membranes were incubated with rabbit anti-TGF-β, IL-4 and β-actin antibodies. After reacting with peroxidase-conjugated secondary antibodies, blots were developed using Hybond-Electrochemiluminescence.

### ELISA

The concentrations of collagens COL1α1and COL1α2 in lung tissue supernatants were determined by ELISA (Uscn Life Science Inc., Hubei, PRC) in duplicate in accordance with manufacturer’s recommendations. The concentrations were corrected for protein concentration. The lowest limit of detection was 0.156 ng/ml.

### Statistical Analysis

Data were presented in Means ± SEM and analyzed using SPSS software. Statistical analyses including paired t-test (between vagotomized and intact lungs) and independence t-test (between different groups) were regarded as significant when p < 0.05.

## Additional Information

**How to cite this article**: Song, N. *et al.* Vagotomy attenuates bleomycin-induced pulmonary fibrosis in mice. *Sci. Rep.* **5**, 13419; doi: 10.1038/srep13419 (2015).

## Figures and Tables

**Figure 1:**
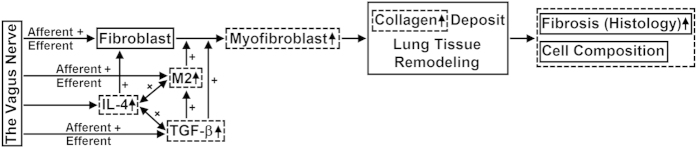
Hypothesis—The vagus nerve promotes fibrotic progression through several pathways. Myofibroblasts at fibrotic lesions are cells responsible for synthesis and deposition of collagen that leads to fibrosis. TGF-β transforms fibroblasts to myofibroblasts. IL-4 serves as an inducer of TGF-β. Activation of the vagal efferent nerve releases acetylcholine, which stimulates fibroblasts by acting on acetylcholine receptors (or by increasing the level of TGF-β) to synthesize collagen. Activation of vagal afferents may produce the axon reflex to release neuropeptides, which polarize macrophages toward a fibrogenic M2 phenotype. Both TGF-β and IL-4 also promote the M2 phenotype. Arrows indicate interaction direction between fibrogenic factors. The dash line squared variables were tested in the protocol to evaluate the effect of vagotomy on bleomycin-induced lung fibrosis. M2, Type 2 (alternatively activated) macrophages; TGF-β, transforming growth factor-β; IL-4, Interleukin-4; α-SMA, α-smooth muscle actin.
